# Downregulation of STK4 promotes colon cancer invasion/migration through blocking β‐catenin degradation

**DOI:** 10.1002/1878-0261.12771

**Published:** 2020-08-25

**Authors:** Cheng‐Han Lin, Tai‐I Hsu, Pei‐Yu Chiou, Michael Hsiao, Wen‐Ching Wang, Yu‐Chia Chen, Jen‐Tai Lin, Jaw‐Yuan Wang, Peng‐Chan Lin, Forn‐Chia Lin, Yu‐Kai Tseng, Hui‐Chuan Cheng, Chi‐Long Chen, Pei‐Jung Lu

**Affiliations:** ^1^ Institute of Clinical Medicine College of Medicine National Cheng Kung University Tainan Taiwan; ^2^ Genomics Research Center Academia Sinica Taipei Taiwan; ^3^ Department of Surgery Chi Mei Medical Center Tainan Taiwan; ^4^ Division of General Surgery Department of Surgery Kaohsiung Veterans General Hospital Kaohsiung Taiwan; ^5^ Division of Urology Department of Surgery Kaohsiung Veterans General Hospital Kaohsiung Taiwan; ^6^ Institute of Clinical Medicine Kaohsiung Medical University Kaohsiung Taiwan; ^7^ Department of Surgery Kaohsiung Medical University Chung‐Ho Memorial Hospital Kaohsiung Taiwan; ^8^ Department of Internal Medicine College of Medicine National Cheng Kung University Hospital National Cheng Kung University Tainan Taiwan; ^9^ Department of Radiation Oncology College of Medicine National Cheng Kung University Hospital National Cheng Kung University Tainan Taiwan; ^10^ Department of Orthopedics Show Chwan Memorial Hospital Changhua Taiwan; ^11^ Department of Orthopedics College of Medicine National Cheng Kung University Hospital National Cheng Kung University Tainan Taiwan; ^12^ Department of Pathology School of Medicine College of Medicine Taipei Medical University Taipei Taiwan; ^13^ Department of Pathology Taipei Medical University Hospital Taipei Medical University Taipei Taiwan; ^14^ Clinical Medicine Research Center College of Medicine National Cheng Kung University Hospital National Cheng Kung University Tainan Taiwan

**Keywords:** colon cancers, STK4, β‐catenin

## Abstract

Mammalian STE20‐like kinase 1 (MST1/STK4/KRS2) encodes a serine/threonine kinase that is the mammalian homolog of *Drosophila* Hippo. STK4 plays an important role in controlling cell growth, apoptosis, and organ size. STK4 has been studied in many cancers with previous studies indicating an involvement in colon cancer lymph node metastasis and highlighting its potential as a diagnostic marker for colon cancer. However, the role of STK4 defect in promoting colon cancer progression is still understudied. Here, we found that STK4 was significantly downregulated in colon cancer and was associated with distal metastasis and poor survival. Furthermore, STK4 knockdown enhanced sphere formation and metastasis *in vitro* and promoted tumor development *in vivo*. We found that STK4 colocalized with β‐catenin and directly phosphorylated β‐catenin resulting in its degradation via the ubiquitin‐mediated pathway. This may suggest that STK4 knockdown causes β‐catenin phosphorylation failure and subsequently β‐catenin accumulation, consequently leading to anchorage‐independent growth and metastasis in colon cancer. Our results support that STK4 may act as a potential candidate for the assessment of β‐catenin‐mediated colon cancer prognosis.

## Graphical abstract

Here, we found that STK4 was significantly downregulated in colon cancer and was associated with distal metastasis and poor survival. STK4 colocalized with β‐catenin and directly phosphorylated β‐catenin resulting in its degradation via the ubiquitin‐mediated pathway. This may suggest that STK4 knockdown causes β‐catenin phosphorylation failure and subsequently β‐catenin accumulation, consequently leading to anchorage‐independent growth and metastasis in colon cancer.

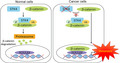

**Abbreviations**
GAPDH: glyceraldehyde 3‐phosphate dehydrogenase
H&E: hematoxylin and eosin
HCC: hepatic cell carcinoma
IHC: immunohistochemistry
PLA: proximity ligation assay
qRT–PCR: quantitative real‐time polymerase chain reaction
Scr: scramble shRNA
STK4: serine/threonine kinase 4
TMA: tissue microarray

## 1 Introduction

Colon cancer is the fourth common cancer in the world [1]. Colon cancer mortality is mainly resulted from lymph node metastasis and distant metastasis [2, 3]. The cancer stem cell theory has been proposed to explain cancer development [4] and metastasis [5, 6]. CD133, CD44, and LGR5 are well‐known cell surface markers for the identification of colorectal cancer stem cells [7]. Downregulation of CD133 [8, 9] or CD44 [10] expression attenuates sphere formation, which is a surrogate assay to assess one of cancer stem cell properties [11]. Based on these studies, targeting stemness‐related genes seems a strategy for colon cancer therapy.

Mammalian STE20‐like kinase 1 (MST1/STK4/KRS2) contains a serine/threonine kinase domain and a SARAH (Sav/Rassf/Hpo) protein interaction domain and is considerably closed to the mammalian homolog of *Drosophila* Hippo [12]. STK4 shares 78% identity in amino acid sequence with STK3 (also called KRS1/MST2) and is originally identified as a pro‐apoptotic cytoplasmic kinase and is important for controlling cell growth, apoptosis, and organ size [13, 14]. In apoptosis, STK4 is cleaved and activated by caspases [15] and then the C‐terminal fragment of STK4 is translocated into the nucleus to enhance chromatin condensation.

The Hippo pathway was first discovered in *Drosophila* [12, 16]. This pathway is associated with the control of organ size by cell proliferation and survival, and its deregulation results in cancer [13]. STK4 is one of the core components of the Hippo pathway [17]. Conditional *STK4* ablation in mice suppresses liver cancer development through promoting YAP phosphorylation [14]. STK4 is also involved in other signaling pathways, for example, AKT signaling [18, 19]. AKT phosphorylates STK4 to block FOXO3 phosphorylation [20]. STK4 is investigated in many cancers, including hepatic cell carcinoma (HCC) [14], malignant gliomas [18], lung cancer [21], head and neck squamous cell carcinoma [22], prostate cancer [23], and colon cancer [24, 25]. Previous studies indicate that STK4 may be a diagnostic marker for colon cancer [24, 26] and liver cancer [27] and be involved in colon cancer lymph node metastasis [24, 28]. Patients with the loss of STK4 show higher tumor stage, vascular invasion, and poor survival [24]. Ablation of STK4/STK3 in mouse liver activates Wnt/β‐catenin signaling and leads to rapid HCC formation [13, 29]. Nuclear β‐catenin overexpression is associated with colon cancer metastasis [30]. However, the role of *STK4* defect in promotion of the progression of colon cancer is still unclear.

Here, our results demonstrated that STK4 was significantly downregulated in tumor tissues and was significantly associated with distal metastasis, disease recurrence, and poor survival. STK4 downregulation promoted sphere formation, tumor development, and metastasis *in vitro* and *in* *vivo*. This may result from the inhibition of STK4‐mediated β‐catenin degradation and consequently enhance β‐catenin expression.

## 2 Methods

### 2.1 Clinical specimens

In this study, two different sets of clinical specimens were used. One set was the screening 167 patients with stage I and stage IV colon cancer, tissue microarray TA40E‐I, which was used to evaluate STK4 expression and clinicopathological factors. Thirty patients, TA35, with normal and tumor pair colon cancer tissues were used to evaluate STK4 expression. The other set was the validating STK4 expression from 93 patients with stage I colon cancer. The experiments were undertaken with the understanding and written consent of each subject. Tissues we used were obtained from Wan Fang Hospital and Taipei Medical University Hospital after Institutional Review Board approval (WFH‐IRB‐99049 and N201701081). The study methodologies confirmed to the standards set by the Declaration of Helsinki.

### 2.2 Immunohistochemistry and quantification of staining intensity

Human and mouse samples were dewaxed and rehydrated. Antigen retrieval was done by incubating the slides in 10 mmol·L^−1^ citric buffer (pH 6.0) and microwaved for 20 min. After blocking, the slides were incubated with primary antibody against STK4 (1 : 200 dilution; Abcam), β‐catenin (1 : 500 dilution; Santa Cruz, Santa Cruze Biotechnology, Dallas, TX, USA), or human mitochondria (1 : 10 000 dilution; Abcam, Cambridge, UK) followed by biotin‐conjugated secondary antibody, polymer‐HRP, and diaminobenzidine tetrahydrochloride (DAB) solution. STK4 staining intensity (*I*) was classified into 4 scores (0, 1, 2, and 3). One hundred cells were counted in each field. The number of positive cells (*P*) at different staining intensities was determined by imagej, https://imagej.nih.gov/ij/ software. Results are scored by multiplying the number of positive cells (*P*) by the intensity (*I*) using the formula *P* × *I*. Maximum score = 300.

### 2.3 Cell culture

Human colon cancer cells CX‐1, H3347, HT29, DLD‐1, LS147T, and SW‐48 were cultured in the culture medium suggested by American Type Culture Collection (ATCC), University Boulevard, Manassas, VA, USA. Cells further underwent identity verification by DNA profiling of short tandem repeat sequences by Mission Biotech (Taipei, Taiwan). All cells were cultured at 37 °C in a humidified atmosphere of 5% CO_2_.

### 2.4 Western blotting

Cell lysates were loaded on SDS/polyacrylamide gel for electrophoresis and transferred to PVDF membranes. Protein expressions were examined after incubation with primary antibodies followed by HRP‐conjugated secondary antibodies and ECL western blotting substrates (Millipore, MilliporeSigma, St. Louis, MO, USA) and detected by X‐ray films. The antibody conditions are listed in Table S1.

### 2.5 *STK4* shRNA lentivirus preparation

*STK4* shRNA was amplified by PCR with the primers: forward (5′‐TGTGTGAAACTGAAACGCCAGTTCAAGAGACTGGCGTTTCAGTTTCACATTTTTGGAAAC‐3′) and reverse (5′‐TCGAGTTTCCAAAAATGTGAAACTGAAACGCCAGTCTCTTGAACTGGCGTTTCAGTTTCACACA‐3′) and constructed into pLentiLox3.7. The pLL3.7‐STK4‐shRNAs and pLL3.7 lentiviral vector‐only lentivirus particles were prepared by co‐transfection with the packaging plasmid SPAX2 and the envelope plasmid MD2G into HEK293T cells.

### 2.6 Plasmid constructs, transfection, and stable clone selection

The full‐length cDNA of human STK4 was amplified using PCR from cDNA libraries with the primer set as follows: forward (5′‐GACAGCGGCCGCATGCGGTGGTAC‐3′) and reverse (5′‐GACACTCGAGTCAGAAGTTTTGTTG‐3′) and constructed into pEGFPC1. STK4‐K59R cells were constructed using site‐directed mutagenesis.

### 2.7 Migration and invasion assays

Cells (1 × 10^5^ cells·mL^−1^) were seeded in 8‐mm‐pore size inserts (BD Biosciences). Serum‐free medium and growth medium containing FBS were added into the upper and lower layers of each insert. For migration assay, cells were incubated at 37 °C for 16–18 h. For invasion assay, the inserts were coated with 1 mg·mL^−1^ of Matrigel Matrix (BD Biosciences, San Jose, CA, USA) and incubated at 37 °C for 10–12 h. Subsequently, the lower side of cells was fixed with methanol and stained with 10% Giemsa (Sigma, MilliporeSigma). The images were captured under the microscopy Olympus IX71 (Olympus, Shinjuku‐ku, Japan), and the cells were counted by imagej.

### 2.8 Animal models

NOD‐SCID mice were obtained from the National Cheng Kung University Laboratory Animal Center and randomly grouped for each animal experiment. All mice were maintained under standard protocols, and the experiment was approved by the Institutional Animal Care and Use Committee, NCKU (IACUC Approval No: 105012). For metastasis experiments, we established orthotopic and intrasplenic injection model. For orthotopic injection, 6‐ to 8‐week‐old male mice were anesthetized with Zoletil 50 by intraperitoneal injection. The cecum would be exteriorized by using abdominal surgery. Following, 1 × 10^6^ CX‐1 scramble and CX‐1 shSTK4 groups were resuspended in 50 μL of medium mixing with 50 μL Matrigel. Mixtures were slowly injected into the cecal wall. Afterward, we would use a cotton stick and pick up and smear 3% iodine at 2 mm from the injection point to avoid seeding of unlikely refluxed tumor cells into the abdominal cavity. After injection, the gut was returned to the abdominal cavity and closed with surgical grapes [31]. For intrasplenic injection, mice were inoculated with CX‐1 with Scr (*n* = 5) or *STK4* shRNA (*n* = 7). 1 × 10^6^ cells were injected into the spleen per mouse. After 4 weeks postinjection, the primary tumor was surgically removed, and the incision was sealed. Mice bearing tumors were imaged by IVIS Imaging System (PerkinElmer, Waltham, MA, USA) every week. At indicated times, mice were sacrificed, and cecum, lung, and liver nodules were counted and further verified by HE and immunohistochemistry (IHC) stain. For xenograft tumor growth, each mouse was subcutaneously inoculated with 1 × 10^3^ or 1 × 10^5^ cells of Scr and *STK4* shRNA stable clones of CX‐1 cells in the left and right flanks, respectively. The tumor size was measured weekly. To assess the outcomes, different investigators rotatably measured the tumor sizes weekly, and the IVIS images were taken by the specialist in National Cheng Kung University Laboratory Animal Center.

### 2.9 Sphere‐forming assay

1 × 10^3^ cells were cultured with DMEM/F12 medium (Gibco, Gaithersburg, MD, USA) containing 20 ng·mL^−1^ rhEGF (PeproTech), 10 ng·mL^−1^ rhbFGF (PeproTech, Rocky Hill, NJ, USA), and N2 supplement (Invitrogen, Waltham, MA, USA) in the ultralow attachment for 7 days. The sphere number was counted by imagej.

### 2.10 Quantitative real‐time PCR

Total RNA isolation and reverse transcription were conducted using the method as described previously [32]. The mRNA expression was analyzed by qPCR. The results were normalized to those of the housekeeping gene glyceraldehyde‐3‐phosphate dehydrogenase (*GAPDH*). Primer sequences are listed in Table S2.

### 2.11 Immunofluorescence assays

1 × 10^4^ cells (DLD‐1) were seeded on coverslips in 12‐well dishes and allowed to attach and grow for 24 h. The following day, pCMV2‐Flag‐STK4 was transfected into DLD‐1 cells. After 4‐h incubation, the transfection medium was replaced by serum‐free medium for 6 h of starvation. The cells were then stimulated with 100 nmol·L^−1^ of insulin for 10 min. After fixation and permeabilization, the cells were incubated with anti‐flag primary antibody and anti‐β‐catenin primary antibody (1 : 50 dilution; Abcam) followed by GFP‐conjugated secondary antibody and rhodamine‐conjugated secondary antibody (1 : 100 dilutions; Abcam).

### 2.12 *In situ* proximity ligation assay

DLD‐1 cells were seeded on coverslips and transfected with Flag‐STK4 construct. After a 12‐h starvation, cells were stimulated with 10% serum for 30 min. PLA was conducted according to the Olink Bioscience protocol using Duolink PLA reagents with minor changes (Olink Bioscience, Uppsala, Sweden). The number of PLA signals was counted using imagej software.

### 2.13 Immunoprecipitation assay

SW48 cells were transfected with GFP and GFP‐STK4 constructs. Cells were treated with 100 nmol·L^−1^ of insulin after a 12‐h starvation period and then lysed with radioimmunoprecipitation assay buffer containing proteinase inhibitors. Co‐IP was conducted using the Catch and Release Reversible Immunoprecipitation System (Upstate).

### 2.14 *In vitro* kinase assay

Cells were transfected with wild‐type STK4 or STK4 mutant plasmids for 24 h to collect total cell lysate. Twenty microlitre of immobilized β‐catenin antibody bead slurry was added to 200 μL cell lysate, incubated with gentle rocking overnight at 4 °C, and then the pellet was washed two times with cell lysis buffer. *In vitro* kinase assays were performed in the kinase buffer: 25 mm Tris (pH 7.5), 5 mm β‐glycerophosphate, 2 mm DTT, 0.1 mm Na_3_VO_4_, and 10 mm MgCl_2_. Kinase reactions were performed 30 min at 30 °C in buffer supplemented with 10 μm ATP and 2 μCi [γ‐32P] ATP. Reactions were stopped by the addition of 2× sample buffer and heating to 100 °C. Reaction products were visualized by SDS/PAGE followed by autoradiography or subjected to western blotting analysis.

### 2.15 Luciferase reporter assay

The luciferase activity was measured as described previously [33]. Cells were co‐transfected with indicated reporter and expressing plasmids. At 24 h after transfection, luciferase activity was measured by Luminoskan Ascent Microplate Luminometer (Thermo Labsystems Inc., Franklin, MA, USA).

### 2.16 Statistical analysis

The statistical analysis was performed using prism5 and spss17 software (New York, NY, USA). Disease‐free survival rate and overall survival rate were estimated using Kaplan–Meier methods and compared among age groups using log‐ranking tests. Cox proportional hazards regression models with univariate and multivariate analyses were summarized with hazard ratios and 95% confidence intervals. Data from all three independent experiments were presented as the mean ± SD and analyzed by unpaired two‐tailed Student's *t*‐test. For all tests, *P* < 0.05 was used to define statistical significance (*P < 0.05, **P < 0.01, ***P < 0.001, ****P < 0.0001).

## 3 Results

### 3.1 STK4 defects are correlated with tumor progression and poor survival rate in colon cancer patients

**Fig. 1:**
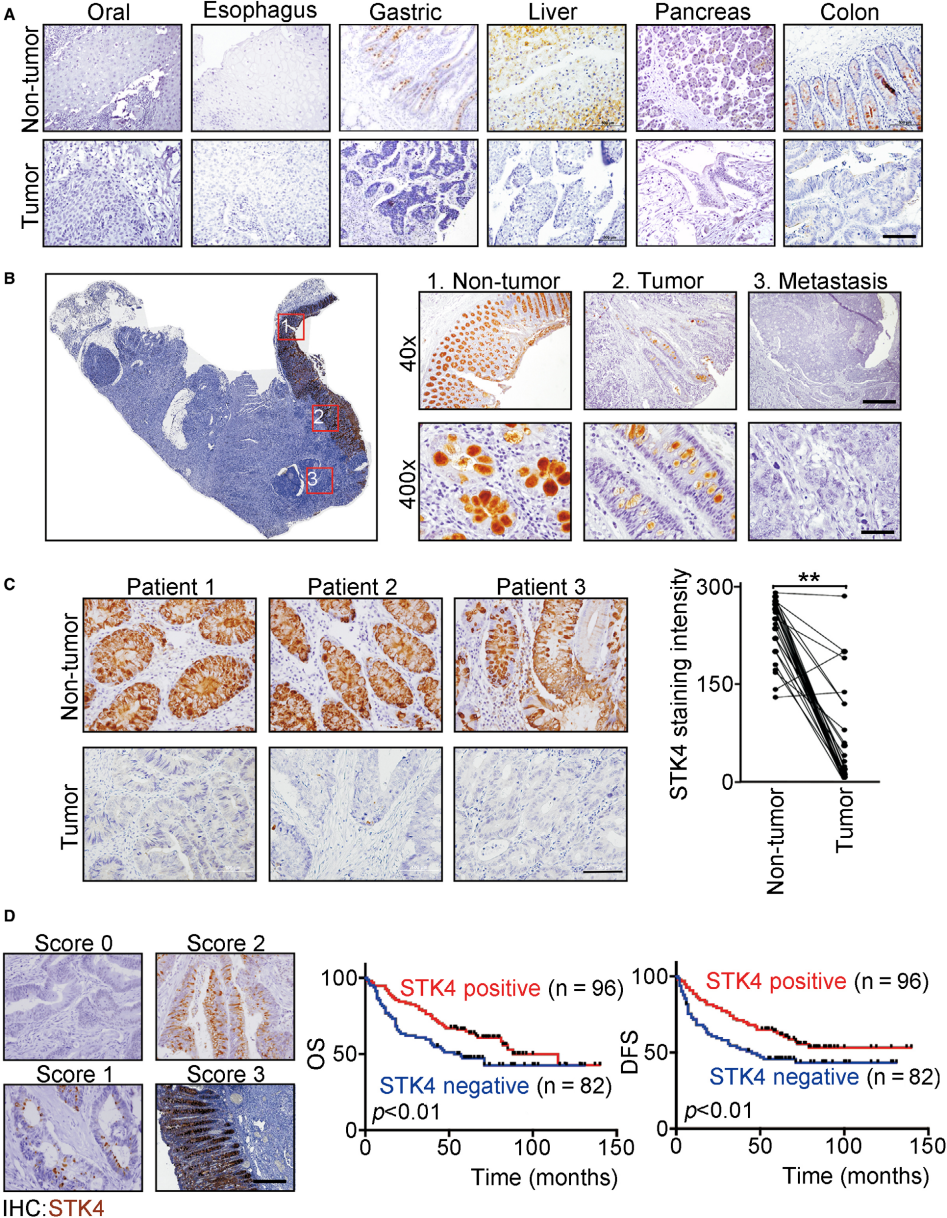
Loss of STK4 expression is associated with cancer progression and poor survival rate in colon cancer patients. (A) STK4 expression (brown) in nontumor and tumor areas of the digestive system was assessed by IHC staining. The scale bar is 500 μm. (B) The left panel represents a whole tissue image. The right panels represent three different areas were picked from the left panel (no. 1, 2, and 3) under different magnifications (40× and 400×). The numbers (left and right panels) indicate STK4 expression (brown) in nontumor, tumor, or metastatic tissues. The colorectal cancer specimen was counterstained with hematoxylin. The scale bar is 500 μm (40×) and 50 μm (400×). (C) The left panels show that STK4 was highly expressed in nontumor tissues (upper panels) of three patients but lost in tumor tissues (lower panels). The right panel shows the quantification of STK4 staining intensity between nontumor and tumor parts (*n* = 30); **P < 0.01. Unpaired two‐tailed *t‐*test. Data are expressed as mean ± SD. The scale bar is 50 μm. (D) The left panels show the classification of STK4 staining intensity into 4 scores. The middle and right panels demonstrate that STK4 expression is associated with patient survival that was analyzed by using Kaplan–Meier curves. The scale bar is 100 μm.

To determine the pattern of STK4 expression in human normal and tumor tissues, we performed IHC staining. The normal tissues of colon and liver displayed intense STK4 cytoplasmic immunoreactivity. However, the loss of STK4 expression was frequently observed in various types of tumors (Fig. 1A). Quantitation of STK4 expression in nontumor and tumor tissues showed that STK4 was preferentially expressed in nontumor tissues of colon, liver, and stomach when compared to breast and lung cancer (Fig. S1). To validate whether the level of STK4 expression in colon decreased within disease progression, IHC was performed in the whole tissue of colon containing the benign section to primary cancer and its adjacent section. STK4 was highly expressed in the benign section but low or no expression of STK4 in tumor and metastatic tissues (Fig. 1B, right panels). The similar results are also found in Fig. 1C. To investigate the correlation of STK4 expression and patient survival, the scoring criteria of STK4 expression in tumor samples were divided into STK4‐positive (scores 1, 2, and 3) and STK4‐negative (score 0) (Fig. 1D, left panels). Kaplan–Meier analysis indicated that 82 patients with STK4‐negative were significantly associated with poor survival compared to 96 patients with STK4‐positive in both disease‐free survival and overall survival (*P* < 0.01) (Fig. 1D, right panels). The relationship between STK4 expression and clinicopathological factors was further analyzed in 140 colon cancer patients. The STK4 expression levels were defined as score 0 in 62 of 140 cases (44.3%), score 1 in 41 of 140 cases (29.3%), score 2 in 23 of 140 (16.4%), and score 3 in 14 of 140 cases (10%). Under these criteria, patients were stratified into STK4‐positive (scores 1, 2, and 3) and STK4‐negative (score 0) groups. Table 1 showed that loss of STK4 expression was significantly associated with degree of invasion of the intestinal wall (T3‐4; *P* = 0.003), distal metastasis (M1; *P* = 0.003), and disease recurrence (Yes; *P* = 0.003). To validate whether the expression level of STK4 in colon can use as an early diagnosis marker, tissues of 93 patients with stage I colon cancer were collected and IHC was performed. The results indicated that STK4 highly expressed in nontumor sections, but low or no expression of STK4 was found in the benign or tumor tissues (*P* < 0.001) (Fig. S2). Taken together, our data indicated that low STK4 was correlated with poor outcomes in colon cancer patients.

**Table 1:** The relationship between STK4 expression and clinicopathological factors in 140 colon cancer patients. The characteristic numbers of emboli (n=139), perineural (n=139), and recurrence (n=138).

Characteristics	STK4 expression		*P* value
	Negative (*n*)	Positive (*n*)	
Age			
Years (mean ± SD)	68.1 ± 13.7	69.8 ± 12.9	0.617
Gender			
Male	32	42	0.793
Female	30	36	
Tumor status			
T1–2	3	18	0.003**
T3–4	59	60	
Lymph node status			
N0	25	41	0.149
N1–3	37	37	
Distal metastasis status			
M0	46	72	0.003**
M1	16	6	
Emboli			
No	21	42	0.022
Yes	40	36	
Perineural			
No	41	60	0.202
Yes	20	18	
Recurrence			
No	31	58	0.003**
Yes	31	18	

** *P* < 0.01.

### 3.2 STK4 downregulation enhances metastasis *in vitro* and *in vivo* in colon cancer

**Fig. 2:**
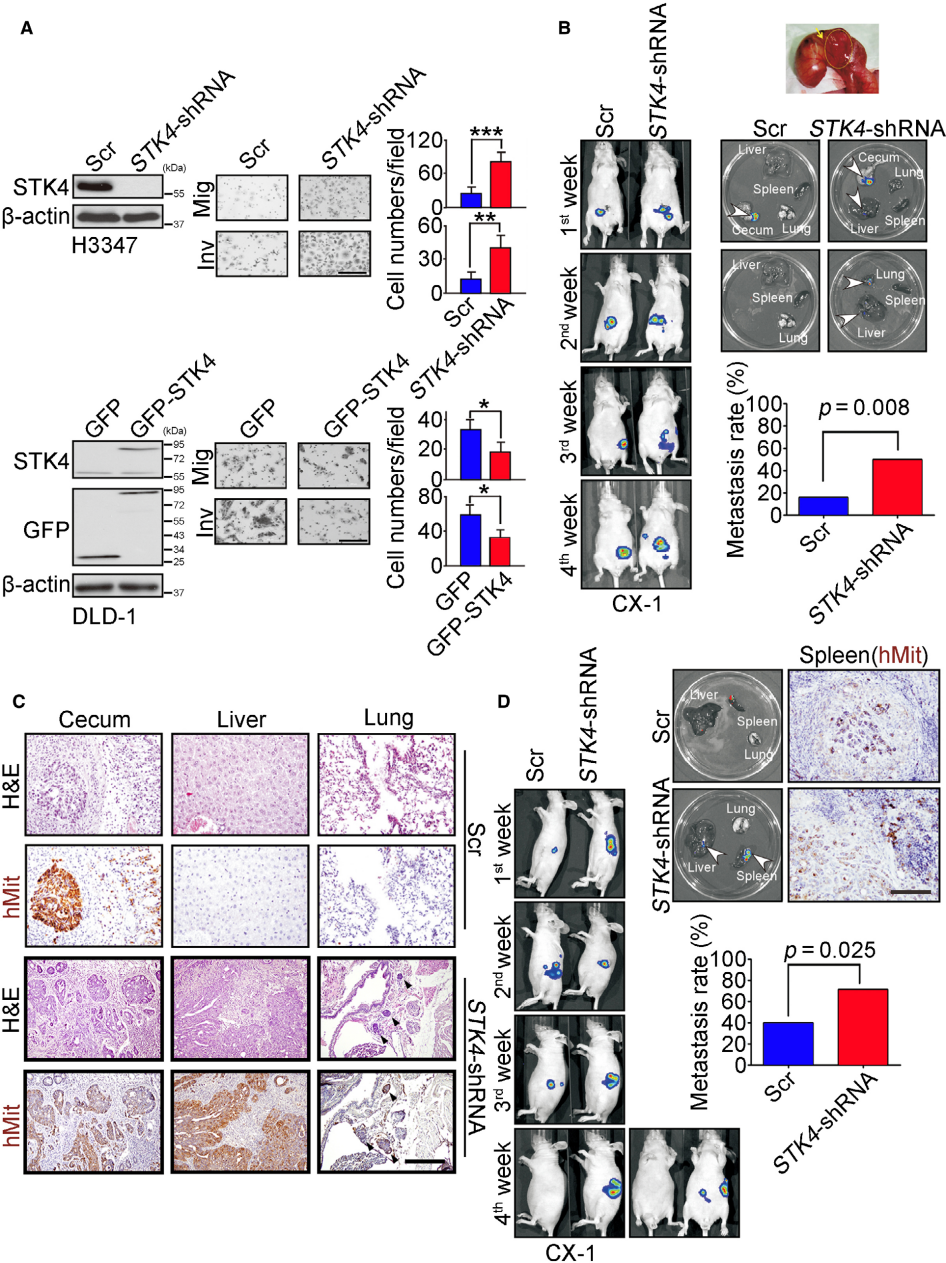
Colorectal cancer cells with STK4 downregulation have high metastatic ability in *in vitro* and *in vivo*. (A) The abilities of migration and invasion of colon cancer cell lines were evaluated by using transwell assay (middle panels). The right panels show the quantification of cell numbers under *STK4* knockdown or overexpression conditions. The left panels indicate the level of STK4 under *STK4* knockdown or overexpression conditions. Data are expressed as mean ± SD. Three independent experiments were performed. In H3347 cells, the migrated cells in the Scr group were 24 ± 12 and in the STK4‐shRNA group were 81 ± 18 (*P* = 0.00006). The invaded cells in the Scr group were 12 ± 4 and in the STK4‐shRNA group were 39 ± 13 (*P* = 0.0062). In DLD‐1 cells, the migrated cells in the GFP group were 34 ± 5 and in the GFP‐STK4 group were 18 ± 4 (*P* = 0.037). The invaded cells in the GFP group were 60 ± 11 and in the GFP‐STK4 group were 33 ± 8 (*P* = 0.041). The scale bar is 200 μm. (Scr, scramble shRNA; Mig, migration; Inv, invasion) (B) The upper panels show the effects of *STK4* knockdown in the metastasis of colon cancer (CX‐1) cells by using the orthotopic mouse model. The middle panels show luciferin activity of different organs imaged by the IVIS system. The lower panel shows the effect of Scr (*n* = 6) or *STK4* knockdown (*n* = 8) in metastasis rate. Chi‐square test was used to compare the metastasis rate in the Scr and STK4 knockdown groups (*P* = 0.008). (C) H&E staining (upper panels) and IHC staining for human mitochondria (lower panels) were used to clarify human cells in different organs from orthotopic microinjection mice. Arrowheads indicated the tumor nodules. Scale bar, 100 μm. (D) The intrasplenic model was the second model to validate the metastasis ability of colorectal cancer cells (Scr group, *n* = 5; *STK4‐*shRNA group, *n* = 6). Chi‐square test was used to compare the metastasis rate in the Scr and STK4 knockdown groups (*P* = 0.025). *P < 0.05, **P < 0.01, ***P < 0.001. Scale bar, 100 μm.

To determine whether STK4 expression involved in colon cancer cells' migration and invasion, colon cancer cells expressing full length of STK4, scramble shRNA (Scr), and *STK4*‐shRNAs were established. STK4 knockdown in H3347 cells significantly increased migration (*P* < 0.001) and invasion (*P* < 0.01) (Fig. 2A, top panels). STK4 overexpression in DLD‐1 cells significantly inhibited migration (*P* < 0.05) and invasion (*P* < 0.05) (Fig. 2A, bottom panels). To further investigated whether STK4 downregulation enhanced metastasis *in vivo*, cells expressing Scr and *STK4*‐shRNAs were orthotopically injected in mice followed by IVIS system to monitor the metastasis. Figure 2B shows that luciferin signal was observed in cecum at the first week. At 4 weeks after cecum injection, lung or liver metastasis was observed in the *STK4*‐shRNA group, while no distal organ metastasis was found in the control group. The quantification of luciferin signal indicated that STK4 knockdown significantly increased colon cancer metastasis rate (chi‐square test, *P* = 0.008). Hematoxylin and eosin (H&E) staining and IHC were performed to confirm the distal metastatic cells derived from human colon cancer cells. The expression of human mitochondria was detected in cecum, liver, and lung tissue sections in the *STK4*‐shRNA group. In the scramble control group, the tumor cells were only observed in the orthotopic tissues (Fig. 2C). Intrasplenic injection of cells expressing Scr and *STK4*‐shRNAs was the other *in vivo* metastasis model used to investigate the liver metastatic ability of colon cancer cells. Figure 2D shows that 4 weeks after intrasplenic injection, liver metastasis was only observed in the STK4 knockdown group. The quantification of luciferin signals indicated that STK4 knockdown significantly increased colon cancer liver metastasis rate (chi‐square test, *P* = 0.025). There was no significant difference in cell proliferation rate after manipulating STK4 expression levels in colon cancer cells (Fig. S3), which was the similar finding shown in Kim's group [34]. According to the above results, our data suggested that the downregulation of STK4 could promote colon cancer metastasis *in vitro* and *in vivo*.

### 3.3 STK4 downregulation enhances the stem cell‐like ability and β‐catenin expression in colon cancer

**Fig. 3:**
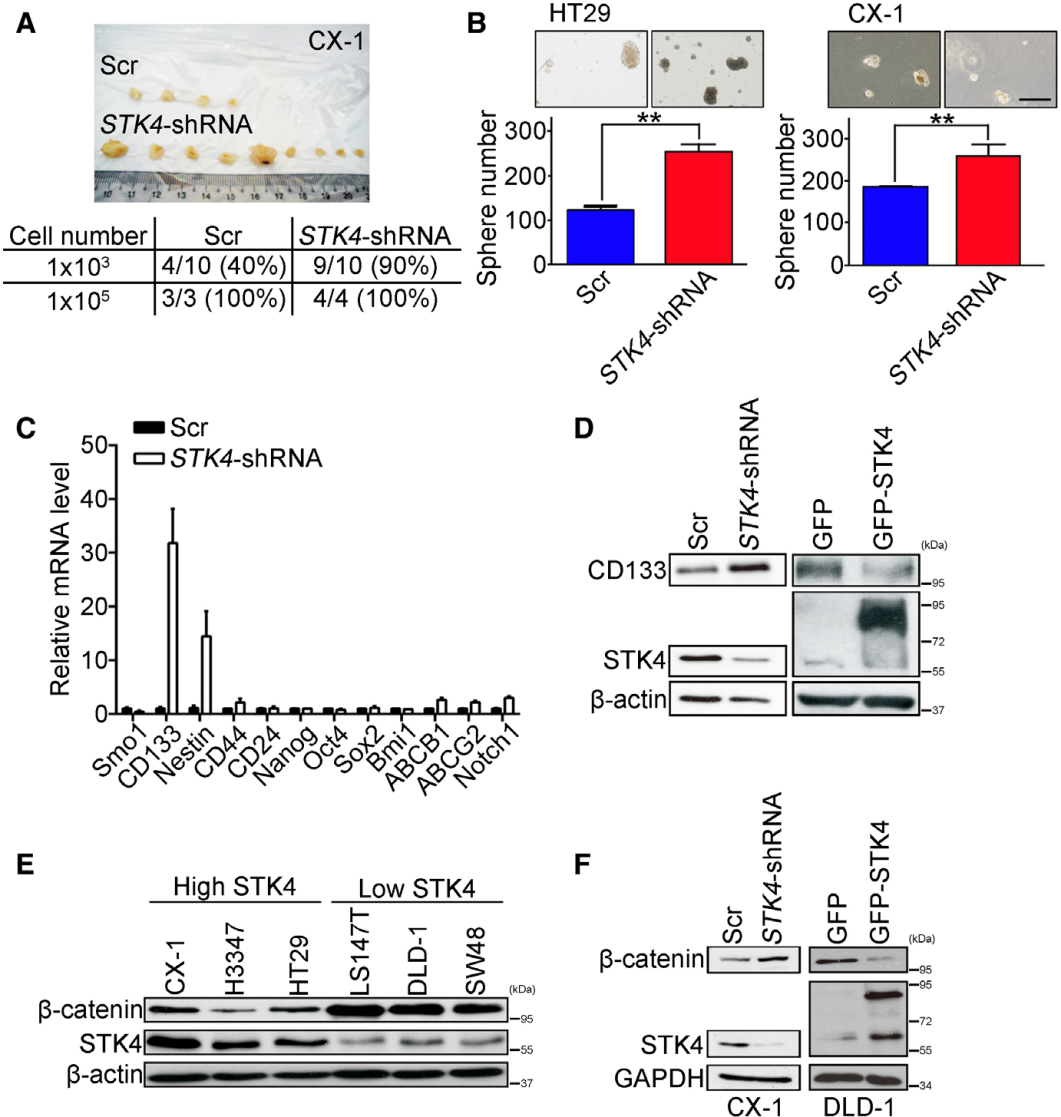
STK4 downregulation enhances the stem cell‐like ability in colon cancer. (A) 1 × 10^3^ or 1 × 10^5^ cells were subcutaneously injected into nude mice (in 1 × 10^3^ group, *n* = 10 both in the Scr and *STK4* shRNA groups; in 1 × 10^5^ group, *n* = 3 in the Scr group and *n* = 4 in the *STK4* shRNA group). The upper panel shows the effect of STK4 knockdown in tumor‐forming ability of 1 × 10^3^ (CX‐1) cells; the lower panel shows the quantification results. (B) 1 × 10^3^ single cells expressing Scr or *STK4* shRNA were cultured in ultralow attachment plate for 7 days. Depicted images (upper panels) show one field per well. A particle in sized over than 50 μm was defined as a sphere. Scale bars, 500 μm. The lower panels show the effect of STK4 knockdown in sphere‐forming ability of colon cancer cells. **P < 0.01, unpaired two‐tailed *t‐*test. Data are expressed as mean ± SD. Three independent experiments were performed to evaluate sphere‐forming ability in STK4 knockdown HT29 and CX‐1 cells. In HT29 cells, the sphere number in the Scr group was 123 ± 19 and 252 ± 33 in the *STK4*‐shRNA group (*P* = 0.0043). In CX‐1 cells, the sphere number in the Scr group was 184 ± 6 and 244 ± 30 in the *STK4*‐shRNA group (*P* = 0.008). (C) Total RNA was extracted from spheres derived (HT29) cells expressing Scr or *STK4* shRNA for the assessment of mRNA expression of stemness and drug‐resistant related genes. All data were normalized against *GAPDH* mRNA level. (Three independent experiments were performed; the results were analyzed by unpaired two‐tailed Student's *t*‐test.) (D) STK4 knockdown or overexpression cells were collected for total cell lysate. The CD133 and STK4 were examined through western blotting. (Three independent experiments were performed.) (E) Adherent cells with high STK4 expression or low STK4 expression were collected for total lysates. The β‐catenin and STK4 were examined through western blotting. (Three independent experiments were performed.) (F) STK4 knockdown or overexpression cells were collected for total cell lysate. The β‐catenin and STK4 were examined through western blotting. (Three independent experiments were performed.)

To investigate whether STK4 knockdown increased colon cancer cells' stemness properties, tumor initiation and sphere‐forming abilities were performed. Nude mice subcutaneously injected with colon cancer cells expressing Scr and *STK4*‐shRNAs were used to evaluate tumor initiation ability. Figure 3A shows that the tumor incidence rate of 1 × 10^3^ cells injected mice was increased in the *STK4*‐shRNA group (90%) compared with the control group (40%). STK4 downregulation significantly increased sphere‐forming ability in HT29 and CX‐1 cells (Fig. 3B). Quantitative real‐time polymerase chain reaction (qRT–PCR) was further used to evaluate the mRNA expression of stemness and drug‐resistant genes after STK4 downregulation. Figure 3C shows that cancer stem cell markers (*CD133*) and *Nestin* in the *STK4*‐shRNA group were increased compared with the control group. The drug‐resistant related genes (*ABCB1* and *ABCG2*) showed no difference after STK4 downregulation. Two ways of demonstrations by overexpression and knockdown of STK4 in colon cancer cells can conclude that STK4 levels were inversely correlated with CD133 expression (Fig. 3D). Previous studies indicate that the Wnt/β‐catenin pathway is involved in self‐renewal of cancer stem cells and metastasis [35, 36, 37]. Furthermore, the loss of STK4 increased β‐catenin expression [14, 36]. To investigate whether STK4 downregulation caused colon cancer stemness and metastasis was associated with β‐catenin expression, six colon cancer cell lines were assessed for STK4 and β‐catenin expression by western blotting. Figure 3E shows that cell lines with high STK4 expression had lower β‐catenin expression compared to cells with low STK4 expression. Overexpression of STK4 in cells decreased β‐catenin expression, and β‐catenin expression in the STK4 knockdown cells was increased (Fig. 3F). According to the above results, our data suggested that downregulation of STK4 promoted colon cancer metastasis may through increase cell stemness properties and through β‐catenin dependent manner.

### 3.4 β‐catenin is directly phosphorylated by STK4 that mediates its degradation and transcriptional activity in colon cancer

**Fig. 4:**
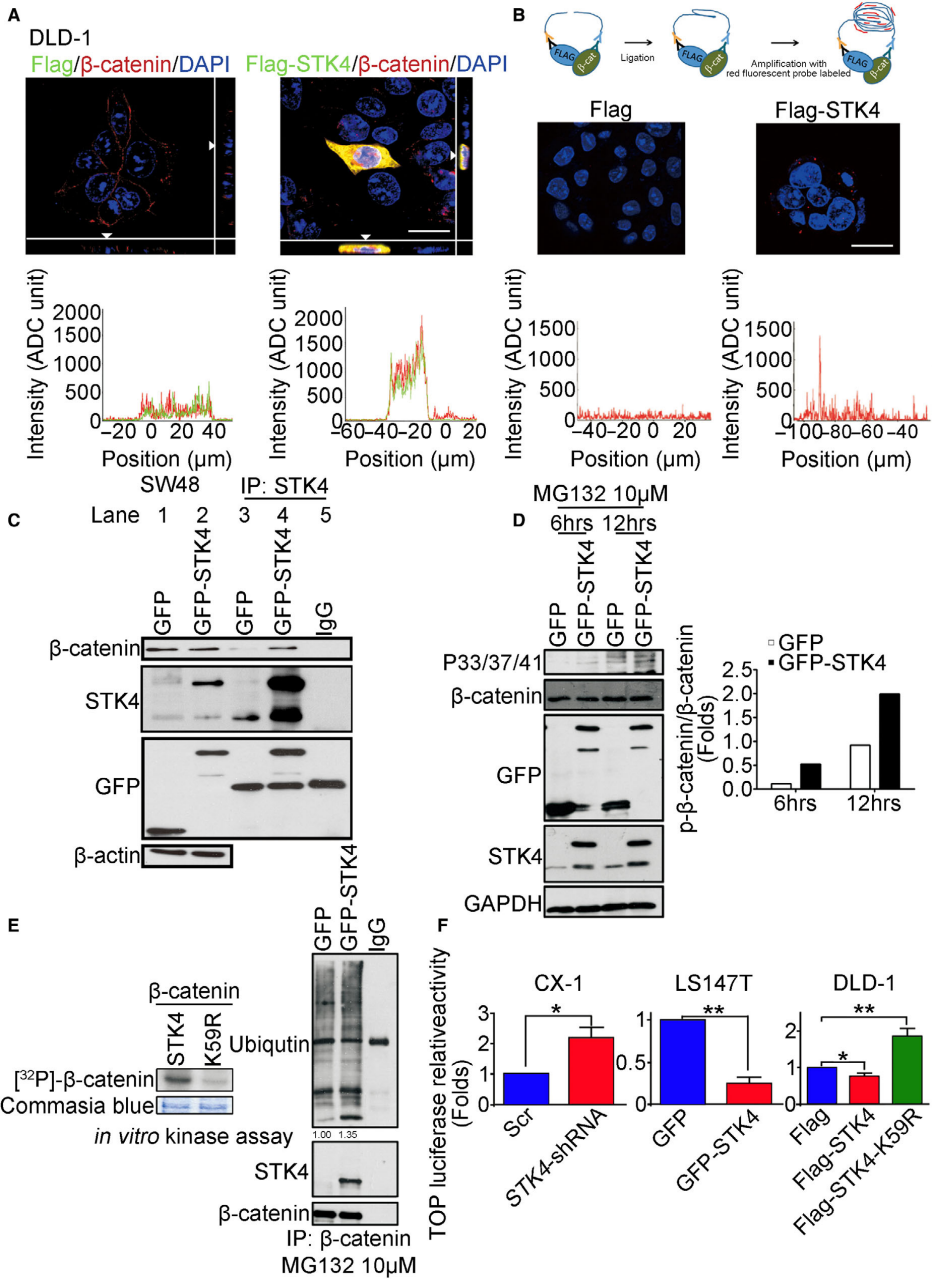
STK4 directly interacts with β‐catenin and mediates its degradation in colon cancer. (A) DLD‐1 cells were transfected with FLAG‐STK4 or FLAG‐empty plasmids (green) for 24 h. These cells were stained for β‐catenin (red) and nuclear DNA (DAPI; blue) and evaluated by confocal microscopy. The yellow area in merged images (right panel) is indicative of STK4/β‐catenin co‐localization. The lower panels show the intensity of STK4 or β‐catenin. Scale bar, 20 μm. (B) A diagram illustrates the principle of PLA assay (upper panel). Merged (right) image shows that STK4 was interacted with β‐catenin in DLD‐1 cells. The lower panels show the intensity of STK4 or β‐catenin. Scale bar, 20 μm. (C) Cells were transfected with GFP‐empty or GFP‐STK4 plasmids. These cells were collected for cell lysates followed by the IP assay (lanes 3, 4, and 5). The STK4, β‐catenin, and GFP were analyzed by western blotting. (Three independent experiments were performed.) (D) DLD‐1 cells were transfected with GFP‐empty or GFP‐STK4 plasmids and cultured in the presence of MG132 for 6 and 12 h. The right panel shows the effect of STK4 overexpression in the ratio of phosphorylated‐β‐catenin/β‐catenin. (Three independent experiments were performed.) (E) Right panel: Cell lysates derived from cells expressing GFP‐STK4 or GFP‐empty vectors were performed IP. The ubiquitin, STK4, and β‐catenin were examined through western blot assays. Left panels: Cells were transfected with wild‐type STK4 or STK4 mutant plasmids for 24 h to collect total cell lysate. Total cell lysates were used to evaluate the ability of STK4‐mediated β‐catenin phosphorylation by *in vitro* kinase assay. The intensity of β‐catenin in STK4 mutant was normalized with the parental STK4 group. (Three independent experiments were performed.) (F) Cells expressing GFP‐STK4, GFP‐empty, *STK4* shRNA, Scr, Flag‐empty, Flag‐STK4, or Flag‐STK4 (K59R) mutant vectors were used to detect β‐catenin protein levels by using TOP‐luciferase assay. Data are expressed as mean ± SD. Three independent experiments were performed. In CX‐1 cells, the fold changes in luciferase activity in the *STK4*‐shRNA group normalized to the Scr group were 2.18 ± 0.67 (*P* = 0.037). In LS147T cells, the fold changes in luciferase activity in the GFP‐STK4 group normalized to the GFP group were 0.24 ± 0.15 (*P* = 0.002). In DLD‐1 cells, the fold changes in luciferase activity in the Flag‐STK4 group normalized to the Flag group were 0.77 ± 0.13 (*P* = 0.038) and in Flag‐STK4‐K59R group normalized to the Flag group were 1.87 ± 0.33 (*P* = 0.012). *P < 0.05, **P < 0.01, ***P < 0.001. Unpaired two‐tailed *t‐*test.

To investigate whether STK4 interacted with β‐catenin *in vitro*, immunofluorescence, *in situ* proximity ligation assay (PLA), and immunoprecipitation assay were performed in colon cancer cells expressing Flag (vector control) and Flag‐STK4. Figure 4A shows that ectopic expression of Flag‐STK4 (green) colocalized with endogenous β‐catenin (red) in the cytoplasm. The quantitation of STK4 and β‐catenin intensity also showed the overlapping signals. As illustrated in Fig. 4B, the STK4‐β–catenin interaction was detected by PLA. The results indicated that Flag‐STK4 interacted with endogenous β‐catenin (Fig. 4B, red spots). Immunoprecipitation assay also showed the physical interaction between GFP‐STK4 and endogenous β‐catenin (Fig. 4C, lane 4). Taken together, these results indicated that STK4 interacted with β‐catenin in colon cancer cells.

In Fig. 3, our data indicated an inverse correlation between STK4 expression and β‐catenin expression in colon cancer cells. To investigate whether STK4 caused β‐catenin phosphorylation, and the phosphorylated β‐catenin was then degraded through proteasomal degradation pathway, colon cancer cells expressing GFP‐STK4 or GFP‐empty plasmids were treated with a proteasome inhibitor (MG‐132) for 6 and 12 h following by western blotting to evaluate the expression of phosphorylated β‐catenin. Figure 4D shows that phosphorylated β‐catenin signal was increased in the GFP‐STK4 group compared with the control group. To further confirm STK4‐mediated β‐catenin phosphorylation, *in vitro* kinase assay was performed in cells expressing STK4 or mutant‐STK4 (K59R). The results showed that phosphorylated β‐catenin signal in the mutant‐STK4 group was decreased (Fig. 4E, left panel). Previous studies indicate phosphorylated β‐catenin is targeted by the F‐box‐containing protein β‐TrCP ubiquitin E3 ligase for its degradation via the ubiquitin–proteasome pathway [38]. Next, we examined whether STK4 overexpression increased phosphorylated β‐catenin degradation. Figure 4E shows ubiquitination in the GFP‐STK4 group was increased compared with the GFP and IgG control groups (right panel). According to the above results, our data suggested that STK4 directly phosphated β‐catenin and consequently led to its degradation via ubiquitin–proteasome pathway.

To examine whether STK4 knockdown affected β‐catenin‐mediated transcriptional activity, the TOP luciferin activity assay was performed in colon cancer cells. Figure 4F shows that STK4 knockdown in CX‐1 cells significantly increased β‐catenin transcriptional activity (*P* < 0.05). STK4 overexpression in LS174t cells reduced β‐catenin transcriptional activity (*P* < 0.01). Furthermore, STK4‐K59R significantly increased β‐catenin transcriptional activity compared with the control group (*P* < 0.01). Thus, STK4 enhanced β‐catenin/TCF‐mediated transcriptional activity.

### 3.5 β‐catenin expression is inversely correlated with STK4 in colon cancer patients

**Fig. 5:**
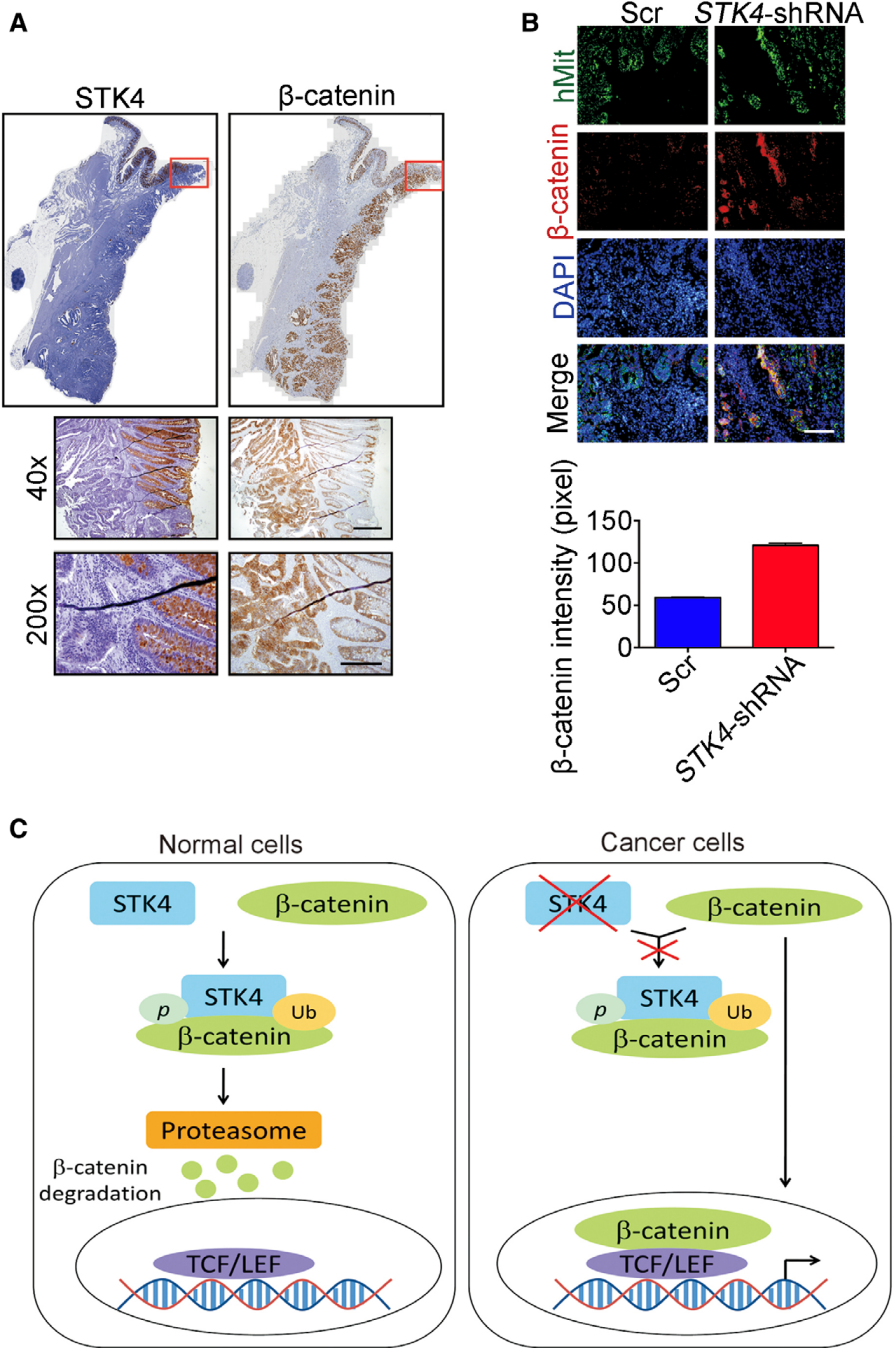
β‐Catenin expression is inversely associated with STK4 expression in colon cancer. (A) Human colon cancer tissues were immunostained with anti‐β‐catenin or anti‐STK4 antibodies, respectively. These samples were counterstained with hematoxylin for nuclei. The upper panels show the whole field of human tissues that expressed STK4 or β‐catenin (brown). The middle and lower panels show the images of different magnification (40× and 200×) from upper panels (red block). Scale bars, 500 μm. (B) Immunofluorescence was used to clarify human cells in mouse colon tissue from orthotopic microinjection mice. Antibody combinations: anti‐human mitochondria (green) and anti‐β‐catenin (red) antibodies. Subsequently, these tissues were counterstained with DAPI for nuclei and evaluated by fluorescent microscopy (40×). The intensity of β‐catenin of mouse colon tissues was quantified to evaluate the effect of *STK4* knockdown. Data are expressed as mean ± SD. In the Scr group, the β‐catenin intensity was 58.82 ± 0.99 pixels (quantified field numbers = 76). In the STK4‐shRNA group, the β‐catenin intensity was 120.51 ± 3.23 pixels (quantified field numbers = 31). Scale bars, 500 μm. (C) A diagram of the relationship between STK4 and β‐catenin in colon cancer.

To evaluate the expression of β‐catenin in colon cancer patients, IHC was performed. Figure 5A shows that nontumor region of human colon tissues showed high STK4 expression but low β‐catenin expression. The benign region of human colon tissues showed low STK4 expression but high β‐catenin expression (red block). To further investigate whether STK4 downregulation in human colon cancer cells affected β‐catenin expression *in vivo*, mice colons were injected with human colon cancer cells expressing Scr or STK4‐shRNA followed by immunofluorescence analysis to detect the expression of β‐catenin. Figure 5B shows that β‐catenin (red) was significantly increased in STK4 knockdown colon cancer cells (green) compared with the control group. Taken together, our findings showed that STK4 was downregulated in colon tumors and correlated with the poor survival of colon cancer patients. STK4 defect was involved in cancer progression. *In vitro* and *in vivo* studies revealed that STK4 downregulation enhanced metastasis ability and increased β‐catenin expression. In addition, STK4 interacted with β‐catenin and directly phosphorylated β‐catenin that led to its degradation via ubiquitin‐mediated pathway (Fig. 5C).

## 4 Discussion

Previous studies have reported that STK4 has tumor‐suppressive ability [13, 25, 27]. Our study shows that STK4 is frequently defecting in colon cancer and the loss of STK4 may be important in the acquisition of metastatic phenotype of colon cancer. However, the role of STK4 in colon cancer cell metastasis has remained unclear. Our IHC results clearly show that most colon nontumor tissues had significantly higher levels of STK4 expression than other organs. Subsequently, the expression dynamics of STK4 were further examined by using colon cancer TMA with complete follow‐up data. These results confirm that STK4 down‐regulation was frequently found in colon cancer tissue and its down‐regulating expression was correlated with tumor metastasis and advanced clinical stage. This suggests that STK4 downregulation may facilitate colon cancer metastasis. Importantly, our data support that the loss of STK4 in colon cancer is a good indicator of overall survival and disease survival, in agreement with Minoo group's finding [24]; STK4 defect results in the failure of β‐catenin phosphorylation and ubiquitination that may subsequently lead to β‐catenin accumulation and consequently result in colon cancer metastasis.

Metastasis remains the main factor of mortality in patients with colon cancer [2, 3] and is correlated with the ability of anchorage‐independent cell growth [39]. Our data show that shRNA‐mediated STK4 knockdown in colon cancer cells increases the abilities of cell migration and invasion (Fig. 2), tumor formation, and capacities of sphere forming (Fig. 3A,B). However, the ectopic expression of STK4 in DLD‐1 cells substantially inhibits cell motility (Fig. 2). Thus, our data may suggest that STK4 is involved in the regulation of anchorage‐independent growth of colon cancer and its downregulation may consequently promote colon cancer metastasis.

Previous studies have reported that β‐catenin overexpression or downregulation regulates anchorage‐independent cell growth [40, 41]. Our data also found that β‐catenin expression is negatively associated with STK4 levels (Figs 3 and 4), and it can be directly phosphorylated by STK4 at S33/S37/T41 sites that subsequently leads to β‐catenin ubiquitination (Fig. 4). Thus, it implies that STK4 may suppress anchorage‐independent growth of colon cancer via downregulation of β‐catenin.

Gene therapy is an experimental strategy for colon cancer therapy with high efficiency [42]. STK4 acts as a tumor suppressor, and its overexpression inhibits tumor growth [21, 43, 44]. Our data also show that the change in STK4 levels negatively regulates β‐catenin‐mediated cell growth and cancer metastatic ability of colon cancer (Figs 2, 3A, and 5B). Importantly, our data support that the level of STK4 expression in colon can be an early diagnosis marker, in agreement with Yu group's finding [45]. Thus, STK4 may be a new candidate for tumor suppressor gene replacement‐mediated colon cancer therapy.

## 5 Conclusions

In conclusion, STK4 plays an important role through interaction with β‐catenin in colon cancer disease models. Our study has provided insights into the role of STK4 in β‐catenin‐mediated colon cancer. STK4 downregulation enhances tumor growth of colon cancer through blocking β‐catenin degradation. Thus, we propose a mechanism how STK4 regulates β‐catenin degradation between normal cells and cancer cells.

## Conflict of interest

The authors declare no conflict of interest.

## Author contributions

C‐HL, T‐IH, P‐YC, and P‐JL designed experiments. C‐HL, T‐IH, and P‐YC performed experiments and data analyses. C‐HL, T‐IH, MH, and P‐JL interpreted the data. W‐CW, Y‐CC, J‐TL, J‐YW, P‐CL, F‐CL, Y‐KT, and C‐LC collected clinical samples and analyzed the clinical data with software. H‐CC assisted with some of the experiments. C‐HL, T‐IH, P‐YC, and P‐JL wrote the manuscript. All authors discussed the results and contributed to the manuscript.

## Supporting information


**Fig. S1.** Quantification of STK4 expression in non‐tumor and tumor areas of patients with different cancers.

**Fig. S2.** STK4 is highly expressed in the normal tissue but show lower or no expression in tumor tissue in early stage I colon cancer patients.

**Fig. S3.** The effect of STK4 expression in cell proliferation of colon cancer cells.

**Table S1.** List of antibodies used in the study.
**Table S2.** Sequences of primers sets.

## Data Availability

The raw data are available from the corresponding author upon reasonable request.
