# Supplementary figures and images for: Downregulation of STK4 promotes colon cancer invasion/migration through blocking β‐catenin degradation
Source: Mol Oncol. 2020 Aug 25;14(10):2574–88. doi: 10.1002/1878-0261.12771 (PMC7530774; doi:10.1002/1878-0261.12771)

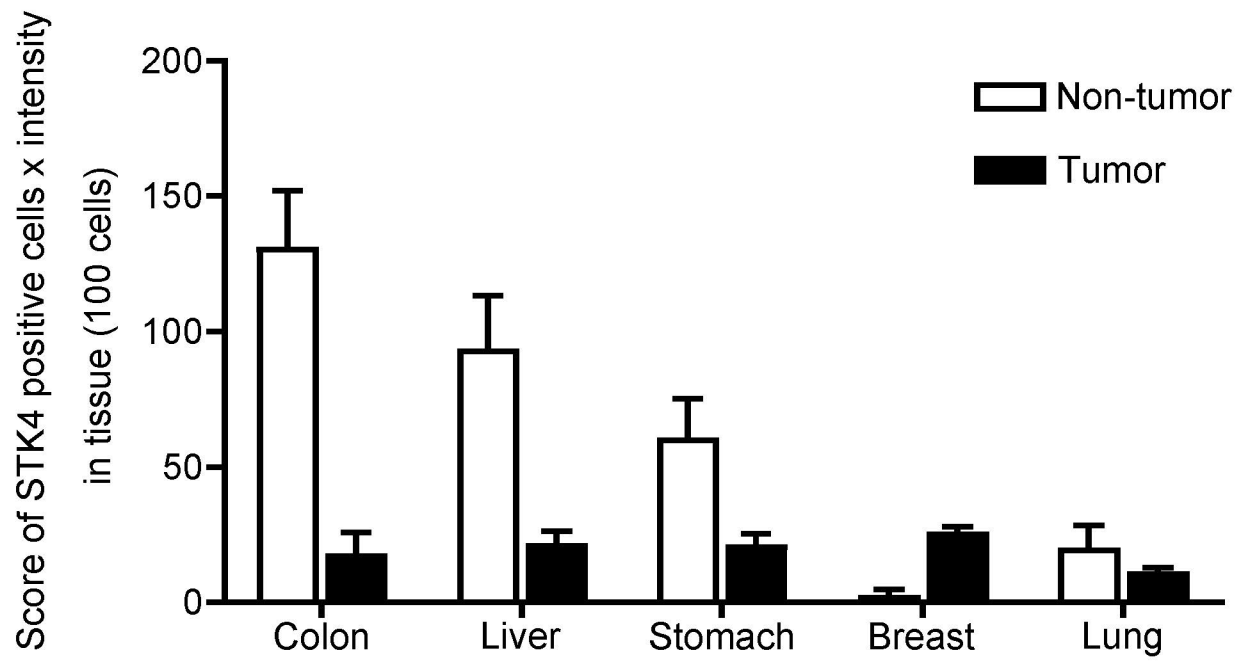

Figure S1. Lin et al

Supplement: Supplementary file 1 — Fig. S1. Quantification of STK4 expression in non‐tumor and tumor areas of patients with different cancers. [file MOL2-14-2574-s001.pdf]

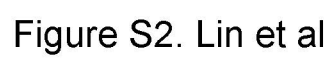

Supplement: Supplementary file 2 — Fig. S2. STK4 is highly expressed in the normal tissue but show lower or no expression in tumor tissue in early stage I colon cancer patients. [file MOL2-14-2574-s002.pdf]

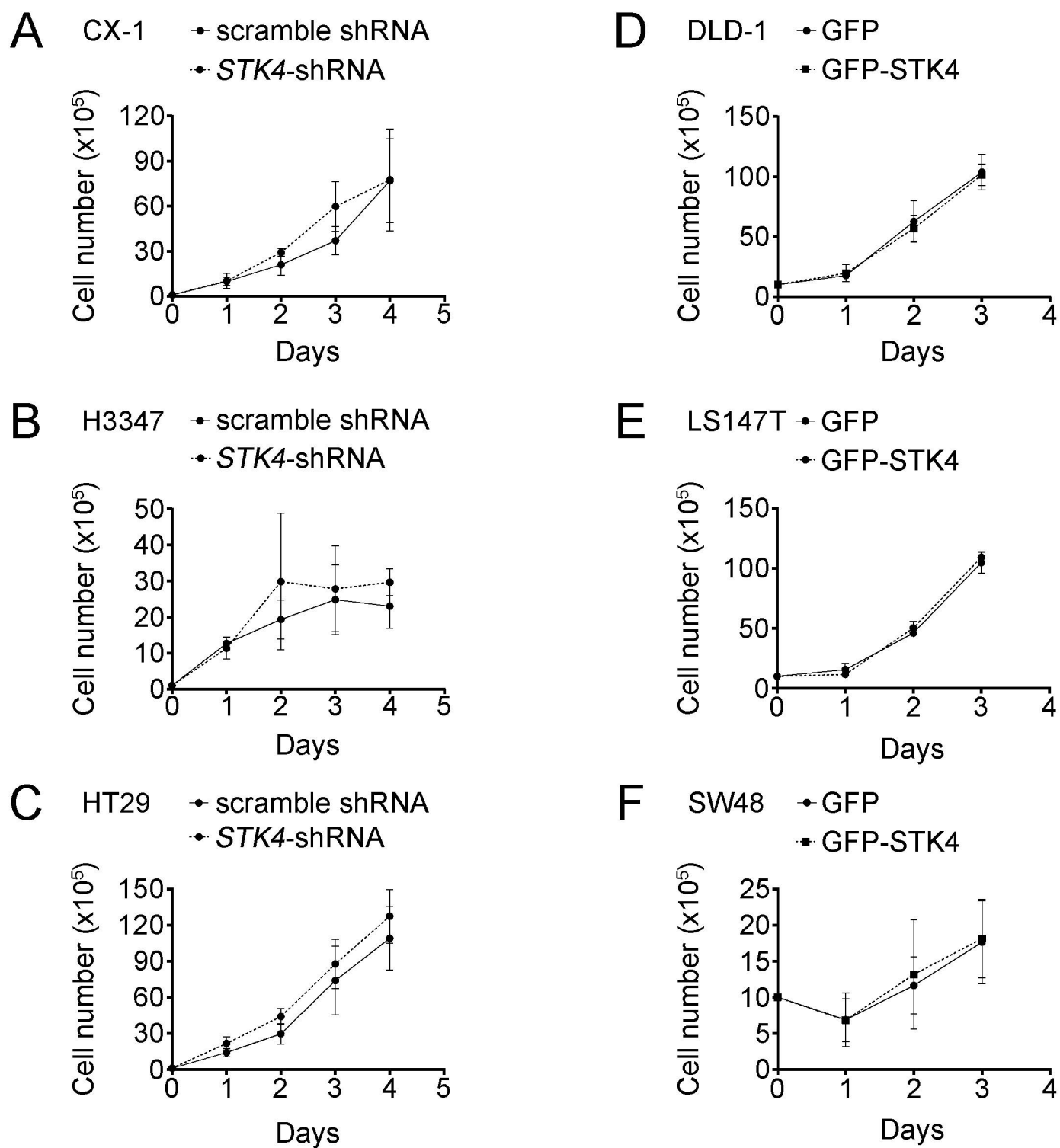

Figure S3. Lin et al

Supplement: Supplementary file 3 — Fig. S3. The effect of STK4 expression in cell proliferation of colon cancer cells. [file MOL2-14-2574-s003.pdf]
