# Supplementary material for: Downregulation of STK4 promotes colon cancer invasion/migration through blocking β‐catenin degradation
Source: Mol Oncol. 2020 Aug 25;14(10):2574–88. doi: 10.1002/1878-0261.12771 (PMC7530774; doi:10.1002/1878-0261.12771)
Supplement: Supplementary file 4 — Table S1. List of antibodies used in the study. Table S2. Sequences of primers sets. [file MOL2-14-2574-s004.docx]

**Supplementary information**

**Downregulation of STK4 promotes colon cancer invasion/migration through blocking β-catenin degradation**

Cheng-Han Lin^1†^, Tai-I Hsu^1†^, Pei-Yu Chiou^1^, Michael Hsiao^2^, Wen-Ching Wang^3^, Yu-Chia Chen^4^, Jen-Tai Lin^5^, Jaw-Yuan Wang^6,7^, Peng-Chan Lin^8^, Forn-Chia Lin^9^, Yu-Kai Tseng^10,11^, Hui-Chuan Cheng^1^, Chi-Long Chen^12,13*^ and Pei-Jung Lu^1,14*^

**Supplementary tables**

| **Supplementary Table S1. List of antibodies used in the study.** | | |  |
| --- | --- | --- | --- |
|  |  |  |  |
| **Western Blot Antibodies** |  |  |  |
| **Antibody** | **Host** | **Catagory** | **Dilution** |
| **Primary antibody** |  |  |  |
| MST1 (STK4) | Rabbit | Cell signaling (3682) | 1:1000 |
| GFP | mouse | Santa Cruz (SC-9996) | 1:10 000 |
| β-actin | mouse | Sigma (A5441) | 1:5000 |
| CD133 | Rabbit | Abcam (ab222782) | 1:1000 |
| β-catanin | Rabbit | GeneTex (GTX101435) | 1:1000 |
| GAPDH | Rabbit | GeneTex (GTX100118) | 1:10 000 |
| Phospho-β-Catenin (Ser33/37/Thr41) | Rabbit | Cell signaling (9561) | 1:1000 |
| **secondary antibody** |  |  |  |
| Goat anti-Mouse IgG (H+L)-HRP |  | Leadgene (20102) | 1:15000 |
| Goat anti-Rabbit IgG (H+L)-HRP |  | Leadgene (20202) | 1:15000 |

| **Supplementary Table S2. Sequences of primers sets** | |
| --- | --- |
|  |  |
| **Primer sequences for qRT-PCR** | |
| **Target gene** | **Primer sequence (5' to 3')** |
| GAPDH | Forward: TGGTATCGTGGAAGGACTCA |
|  | Reverse: AGTGGGTGTCGCTGTTGAAG |
| ABCB1 | Forward: AAATTGGCTTGACAAGTTGTATATGG |
|  | Reverse: CACCAGCATCATGAGAGGAAGTC |
| ABCG2 | Forward: TCATCAGCCTCGATATTCCATCT |
|  | Reverse: GGCCCGTGGAACATA AGTCTT |
| Smo-1 | Forward: TGGTCACTCCCCTTTGTCCTC |
|  | Reverse: GCACGGTATCGGTAGTTCTTC |
| CD133 | Forward: AGTGGCATCGTGCAA ACCTG |
|  | Reverse: CTCCGAATCCATTCGACGATAGTA |
| Nestin | Forward: TGGCTCAGAGGAAGAGTCTGA |
|  | Reverse: TCCCCCATTTACATGCTGTGA |
| CD44 | Forward: AAGACATATACCCCAGCAAC |
|  | Reverse: TTTGCTCCACCTTCTTGACTCC |
| CD24 | Forward: ATGGGCAGAGCAATGGTGGCCA |
|  | Reverse: AGAGTGAGACCACGAAGAGACT |
| Nanog | Forward: AATACCTCAGCCTCCAGCAGATG |
|  | Reverse: TGCGTCACACCATTGCTATTCTTC |
| Oct-4 | Forward: CTTGCTGCAGAAGTGGGTGGAGGAA |
|  | Reverse: CTGCAGTGTGGGTTTCGGGCA |
| Sox-2 | Forward: AACCCCAAGATGCACAACTC |
|  | Reverse: CGGGGCCGGTATTTATAATC |
| Bmi-1 | Forward: TGGAGAAGGAATGGTCCACTTC |
|  | Reverse: GTGAGGAAACTGTGGATGAGGA |
| Notch-1 | Forward: CAGGCAATCCGAGGACTATG |
|  | Reverse: CAGGCGTGTTGTTCTCACAG |
|  |  |

**Supplementary figure legends**

**Figure S1.** Quantification of STK4 expression in non-tumor and tumor areas of patients with different cancers. After IHC staining, histological images of each cancer type were captured the non-tumor and tumor field. The field numbers of each cancer are listed below. 100 cells were quantified in each filed. Classification of STK4 staining intensity (I) into 4 scores (0, 1, 2 and 3). The number of positive cells (P) at different staining intensities was determined by ImageJ software. Results are scored by multiplying the number of positive cells (P) by the intensity (I) using the formula P × I. Maximum score = 300. Data are expressed as mean ±SD. In colon cancer, the score was 131.4±20.4 in non-tumor group (n=9), and the score was 18.3±7.4 in tumor group (n=13). In liver cancer, the score was 93.8±19.7 in non-tumor group (n=5), and the score was 21.9±4.6 in tumor group (n=20). In stomach cancer, the score was 60.9±14.4 in non-tumor group (n=7), and the score was 21.6±3.8 in tumor group (n=21). In breast cancer, the score was 2.8±2.1 in non-tumor group (n=6), and the score was 26.1±1.9 in tumor group (n=34). In lung cancer, the score was 20.3±8.2 in non-tumor group (n=9), and the score was 11.6±1.4 in tumor group (n=26).

**Figure S2.** STK4 is highly expressed in the normal tissue but show lower or no expression in tumor tissue in early stage I colon cancer patients**.** The score of STK4 staining intensity between normal and tumor parts in colon cancer stage I tissues (n=93); **** indicates *p*<0.0001. Unpaired two-tailed *t* test.

**Figure S3.** The effect of STK4 expression in cell proliferation of colon cancer cells. For cell proliferation analysis, 1×10^5^ cells were infected with Scr, *STK4*-shRNA, GFP, and GFP-STK4 plasmids separately. Cells stably expressing *STK4*-shRNA or Scr were cultured for four days; cells expressing STK4 or empty vectors were culture for three days. The cell number of colon cancer was assessed by MTT assay. The left panels (A, B and C) represent the effect of STK4 knockdown in cell growth (n=6); the right panels (D, E and F) represent the effect of STK4 overexpression in cell growth (n=3). Data are expressed as mean ±SD. In CX-1 cells, the cell numbers in Scr group were 9.8±3.0 x10^5^, 21.2±8.9 x10^5^, 37±11.7 x10^5^, 76±34.5 x10^5^ at day1, day2, day3, day4, respectively. In *STK4*-shRNA group were 10.3±8.1 x10^5^, 29.2±4.4 x10^5^, 59.5±9.0 x10^5^, 79.5±45.6 x10^5^ at day1, day2, day3, day4, respectively. In H3347 cells, the cell numbers in Scr group were 12.6±1.9 x10^5^, 19.3±3.4 x10^5^, 24.9±5.5 x10^5^, 22.7±10.3 x10^5^ at day1, day2, day3, day4, respectively. In *STK4*-shRNA group were 11.2±5.5 x10^5^, 29.7±27.6 x10^5^, 27.6±4.8 x10^5^, 29.6±5.3 x10^5^ at day1, day2, day3, day4, respectively. In HT29 cells, the cell numbers in Scr group were 13.9±4.1 x10^5^, 29.5±10.5 x10^5^, 73.8±36.7 x10^5^, 109.2±30.6 x10^5^ at day1, day2, day3, day4, respectively. In *STK4*-shRNA group were 21.6±5.8 x10^5^, 43.8±6.8 x10^5^, 87.5±24 x10^5^, 127.3±22 x10^5^ at day1, day2, day3, day4, respectively. In DLD-1 cells, the cell numbers in GFP group were 17.8±0.7 x10^5^,62.7±17.3 x10^5^, 103.8±15.2 x10^5^, at day1, day2, day3, respectively. In GFP-STK4 group were 19.7±7 x10^5^, 57.1±11.1 x10^5^, 101.5±9.1 x10^5^ at day1, day2, day3, respectively. In LS147T cells, the cell numbers in GFP group were 15.5±5.3 x10^5^, 46±28.3 x10^5^, 104.8±8.8 x10^5^, at day1, day2, day3, respectively. In GFP-STK4 group were 11.5±1.8 x10^5^, 50.3±5.3 x10^5^, 109.3±4.6 x10^5^ at day1, day2, day3, respectively. In SW48 cells, the cell numbers in GFP group were 6.9±3.7 x10^5^, 11.6±4 x10^5^, 17.6±5.8 x10^5^, at day1, day2, day3, respectively. In GFP-STK4 group were 6.8±3 x10^5^, 13.2±7.6 x10^5^, 18.1±5.5 x10^5^ at day1, day2, day3, respectively.
